# OBESITY AND SEVERE STEATOSIS: THE IMPORTANCE OF BIOCHEMICAL EXAMS AND SCORES

**DOI:** 10.1590/0102-672020210002e1626

**Published:** 2022-01-31

**Authors:** Miller Barreto de Brito e SILVA, Francisco TUSTUMI, Anna Carolina Batista DANTAS, Barbara Cristina Jardim MIRANDA, Denis PAJECKI, Roberto DE-CLEVA, Marco Aurelio SANTO, Sergio Carlos NAHAS

**Affiliations:** 1 Department of Gastroenterology, Faculty of Medicine, University of São Paulo - USP - São Paulo - SP, Brazil.

**Keywords:** Fatty Liver, Nonalcoholic Fatty Liver Disease, Obesity, Bariatric Surgery, Fígado Gorduroso, Hepatopatia Gordurosa não Alcoólica, Obesidade, Cirurgia Bariátrica

## Abstract

Nonalcoholic fatty liver disease (NAFLD) is considered a public health problem, mainly in severely obese patients.

**OBJECTIVE:**

The aim of the present study was to investigate different biochemical-based scores available and determine which one could best serve as an NAFLD predicting tool in a severely obese population.

**METHODS:**

This was a cross-sectional study involving severely obese patients. All patients were evaluated with serum laboratory parameters for 1 week before biopsy, and all patients were treated with intraoperative liver biopsy, during bariatric surgery.

**RESULTS:**

A total of 143 severely obese patients were included. The median body mass index (BMI) was 48 kg/m^2^ (35-65). Diabetes mellitus was present in 36%, and steatosis was present in 93% (severe steatosis in 20%). Only aspartate transaminase (AST) to platelet ratio index (APRI=0.65 (95% CI: 0.55-0.8) and homeostatic model assessment for insulin resistance (HOMA-IR=0.7 (95% CI: 0.58-0.82) showed significant capacity for the prediction of severe steatosis. Hepatic steatosis index (HSI), NAFLD fibrosis score (NAFLDS), alanine aminotransferase (ALT)/AST, and fibrosis-4 (FIB-4) were not able to correctly predict severe steatosis on liver biopsy. APRI showed high specificity of 82% and low sensitivity of 54%. In contrast, HOMA-IR showed high sensitivity of 84% and low specificity of 48%.

**CONCLUSIONS:**

NAFLDS, FIB-4, AST/ALT, and HSI have no utility for the evaluation of severe steatosis in severely obese patients. Diabetes and insulin-resistance-related biochemical assessments, such as HOMA-IR, can be used as good screening tools for severe steatosis in these patients. APRI score is the most specific biochemical diagnostic tool for steatosis in severely obese patients and can help clinicians to decide the need for bariatric or metabolic surgery.

## RESUMO - RACIONAL

A doença hepática gordurosa não-alcoólica já é considerada um problema de saúde pública, principalmente em pacientes com obesidade severa.

**OBJETIVOS:**

O objetivo do presente estudo foi investigar os diferentes escores de bioquímiosa disponíveis e determinar qual deles poderia servir melhor como uma ferramenta de avaliação da NAFLD em uma população de obesos.

**MÉTODOS:**

Este é um estudo transversal de pacientes obesos. Todos os pacientes foram avaliados com parâmetros laboratoriais séricos 1 semana antes da biópsia e todos os pacientes foram submetidos a biópsia hepática intra-operatória, durante a cirurgia bariátrica.

**RESULTADOS:**

Cento e quarenta e três pacientes obesos foram incluídos. Apenas APRI (0,65; IC 95%: 0,55 a 0,8) e HOMA-IR (0,7; IC 95%: 0,58 a 0,82) mostraram capacidade significativa de predição de esteatose grave. HSI, NALFDS, ALS / AST e FIB-4 não foram capazes de prever corretamente esteatose grave na biópsia hepática. APRI mostrou alta especificidade (82%) e baixa sensibilidade (54%). Em contraste, o HOMA-IR apresentou alta sensibilidade (84%) e baixa especificidade (48%).

**CONCLUSÃO:**

O NALFDS, FIB-4, AST / ALT e HSI não têm utilidade para avaliação de esteatose grave em pacientes com obesidade severa. Diabetes e avaliação bioquímica relacionada à resistência à insulina, como o HOMA-IR, podem ser empregados como boas ferramentas de rastreamento para esteatose grave em tais pacientes. O escore APRI é a ferramenta diagnóstica bioquímica mais específica para esteatose em pacientes com obesidade severa e pode ser empregado, por equipes médicas, para auxiliar na indicação de cirurgia bariátrica ou metabólica.

## INTRODUCTION

Obesity and overweight are increasing worldwide, and several obesity-related comorbidities are also on the rise^19^. The obese patient has a higher risk for metabolic disorders, including type 2 diabetes mellitus, hypertension, dyslipidemia, and chronic liver disease^8^.

Nonalcoholic fatty liver disease (NAFLD) is a clinical condition characterized by excessive lipid deposits in hepatocytes, without significant ethanol intake, in the absence of other etiologies of liver diseases^14^. Currently, NAFLD occurs in a range of 30-100% in obese adults^18^. In recent years, NAFLD has become the most common cause of liver disease in the Western world and it is already considered a public health problem^8^. NAFLD can progress to steatohepatitis (nonalcoholic steatohepatitis [NASH]), fibrosis, cirrhosis, and potentially to end-stage liver failure or hepatocellular carcinoma (HCC)^22^. In the United States, NASH is the fastest growing reason for liver transplantation^21^.

Although the major risk factors for NAFLD are well established (e.g., age >50 years, obesity, insulin resistance, diabetes mellitus, and metabolic syndrome), the pathological mechanisms by which each of these risk factors causes NAFLD progression to NASH and cirrhosis are unclear^5^. Patients with NAFLD are usually asymptomatic until the condition progresses to liver cirrhosis, which makes early diagnosis challenging^1^.

Liver biopsy remains as the gold-standard diagnostic procedure to detect liver abnormalities compatible with NAFLD and NASH^1^. The main histopathological features of NASH are a combination of steatosis, hepatocyte ballooning, and lobular inflammation. However, performing a liver biopsy in an obese patient has several drawbacks: it is an invasive procedure, with risks of bleeding and bile leaks, and involves the possibility of sampling error^1^. This fact has motivated the development of alternative noninvasive methods, which have been extensively investigated lately. Applying the noninvasive test to predict severe steatosis in severely obese patients could help clinicians to manage these patients, including helping them to decide the need for bariatric or metabolic surgery.

There are several clinical scores primarily based on biochemical markers that can be used to evaluate liver status in severely obese patients^7^ The hepatic steatosis index (HSI) is a screening tool for diagnosing steatosis^15^. There are other liver biochemical scores such as fibrosis-4 (FIB-4)^25^, NAFLD fibrosis score (NAFLDS)^2^, aspartate transaminase/alanine aminotransferase (AST/ALT) ratio^16^, and AST to platelet ratio index (APRI)^27^. These scores are used for estimating liver fibrosis, which occurs in most types of chronic liver diseases. Insulin-resistance index (e.g., homeostatic model assessment for insulin resistance [HOMA-IR])^24^ is another biochemical score used for screening insulin resistance, which is usually associated with steatosis^28^. The summary of these formulas is given in Table 1.

**Table 1 -:** Summary of the formulas used in biochemical-based scores for the evaluation of liver status.

NAFLD score	$NAFLD\;score=\;-1.675\;+\;(0.037*age\;\left[years\right])\;+\;(0.094*BMI\;\left[kg/m^{2}\right])\;+\;(1.13*IFG/diabetes\;\left[Yes\;=\;1,\;no\;=\;0\right])\;+\;(0.99*AST/ALT\;ratio)\;-\;(0.013*platelet\;count\;\left[\times 10^{9}/L\right])\;-\;(0.66*albumin\;\left[g/dl\right])$
HOMA-IR	(HOMA-IR): Glycemia (mmol) × Insulin (IU/ml) ÷ 22.5
FIB-4 score	FIB-4 score = (Age × AST) / (Platelets × √(ALT))
HSI score	HSI = 8 × ALT/AST + BMI (+2 if type 2 diabetes yes, +2 if female)
APRI score	$APRI\;=\;(AST\;in\;IU/L)/(AST\;Upper\;Limit\;of\;Normal\;in\;IU/L)/(Platelets\;in\;10^{9}/L)$
AST/ALT ratio	AST/ALT = ratio

APRI, aspartate transaminase to platelet ratio index; AST/ALT, aspartate transaminase/alanine aminotransferase; BMI, body mass index; FIB-4, fibrosis-4; HIS, hepatic steatosis index; HOMA-IR, homeostatic model assessment for insulin resistance; IFG, impaired fasting glycemia; NAFLDS, nonalcoholic fatty liver disease fibrosis score.

However, until the present time, there is no consensus on which is the best way to evaluate a severely obese patient before considering bariatric or metabolic surgery. Previous reports showed that APRI score is associated with advanced fibrosis in obese patients^9^ However, a suitable biochemical evaluation for severe steatosis, which could help clinicians to properly manage the severely obese patients, is poorly debated.

The aim of the present study was to investigate different biochemical-based scores available and determine which one could best serve as a severe steatosis predicting tool in a severely obese population.

## METHODS

### Participants

This is a cross-sectional study, assessing the diagnostic performance of different NAFLDS based on biochemical analysis for the prediction of severe steatosis. Only severely obese patients were included with an indication for bariatric surgery. Patients of a single institute, from the period 2012 to 2019, were selected, with body mass index (BMI) = 40 kg/m^2^ or = 35 kg/m^2^ with obese-related comorbidities. Only adults were included (>18 years). Exclusion criteria were alcohol abuse (self-reported alcohol intake >40 g/day for men and 20 g/day for women), viral or other known causes of chronic liver disease, pregnancy, and previous history of liver transplantation.

Approval for this study was given by the local ethics committee, which waived the need for informed consent.

### Biochemical analysis

All patients were evaluated with serum laboratory parameters for 1 week before the biopsy. These include liver function tests, serology for hepatitis C and B virus, serum beta-human chorionic gonadotropin, ALT, AST, albumin, cholesterol, triglyceride, glycated hemoglobin A1c, fasting plasma glucose, insulin, C-peptide, and complete blood count.

### Biochemical scores

Based on the biochemical analysis, the following scores were estimated: AST to platelet ratio index (APRI), NAFLDS, FIB-4, AST/ALT ratio, HSI, and HOMA-IR.

### Gold-standard test

All patients were treated with intraoperative liver biopsy during bariatric surgery. The biopsy was performed by four surgeons using a 16-gauge Tru-Cut (Care-151 Fusion; Vernon Hills, IL, USA). Every biopsy specimen was evaluated by senior pathologists and classified according to the proportion of fat-replete hepatic cells, following the NASH Clinical Research Network scoring system as normal steatosis (<5%), mild steatosis (5-33%), moderate steatosis (33-66%), or severe steatosis (>66%).

### Statistical analysis

Statistical analysis of the data was performed using STATA version 16.0 (StataCorp LLC, College Station, TX, USA). The data measured were described as median and range. Categorical variables were described using frequency and percentage. The level of significance adopted was 5%. The diagnostic performance of each score for the prediction of severe steatosis (>66% of fat-replete hepatic cells) was evaluated by the receiver operating characteristic (ROC) curve. The maximum Youden’s index was used to determine the best cutoff point for each score. The area under curve (AUC) was measured for each score, and a test for the equality of the AUCs was made using an algorithm suggested by DeLong^9^. ROC regression analysis was performed to identify the influence of covariates.

## RESULTS

### Baseline characteristics

A total of 143 severely obese patients were included in this study. The median age was 43 years old (19-68), with female predominance (77%). The median BMI was 48 kg/m^2^ (35-65). Diabetes mellitus was present in 36% and steatosis was present in 93% (severe steatosis in 20%). The baseline characteristics are given in Table 2.

**Table 2 -:** Baseline characteristics of the included patients.

N			143
	Median	Min	Max
Age (years)	43	19	68
BMI (kg/m^2^)	48	35	65
			%
Female			77
Diabetes			36
Dyslipidemia			24
Steatosis			
0			7
1			40
2			33
3			20
NAS score			
0-2			21
3-4			45
5-8			34

BMI, body mass index; NAFLD, nonalcoholic fatty liver disease; NAS, NAFLD Activity Score.

### Diagnostic performance

**Figure 1 -:**
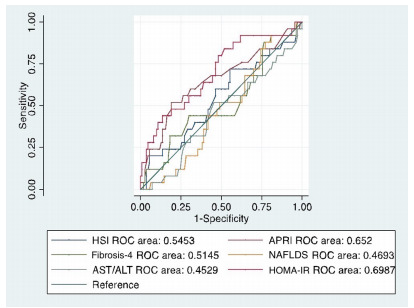
Receiver operating characteristic curves for each score*.*

All scores were evaluated using the ROC curve and the AUC. Only APRI (0.65; 95% CI: 0.55-0.8) and HOMA-IR (0.7; 95% CI: 0.58-0.82) showed significant capacity for the prediction of severe steatosis. HSI, NAFLDS, ALT/AST, and FIB-4 were not able to correctly predict severe steatosis on liver biopsy (see Figure 1 and Table 3).

**Table 3 -:** Area under the curve of different scores.

	AUC	Standard error	95% CI	
			Lower	Upper
HSI	0.55	0.064	0.42	0.67
APRI	0.65	0.063	0.55	0.8
NAFLDS	0.47	0.058	0.35	0.58
ALT/AST	0.45	0.061	0.32	0.56
HOMA-IR	0.7	0.061	0.58	0.82
FIB-4	0.51	0.065	0.34	0.65

APRI, aspartate transaminase to platelet ratio index; AST/ALT, aspartate transaminase/alanine aminotransferase; CI, confidence interval; FIB-4, fibrosis-4; HIS, hepatic steatosis index; HOMA-IR, homeostatic model assessment for insulin resistance; NAFLDS, nonalcoholic fatty liver disease fibrosis score.

**Figure 2 -:**
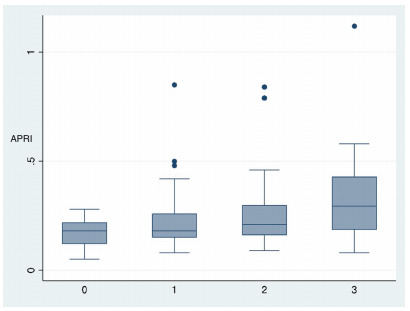
Boxplot analysis for APRI score and the grade of steatosis determined by biopsy, according to the NASH Clinical Research Network (NASH-CRN) scoring system. 0: normal steatosis (<5%); 1: mild steatosis (5-33%); 2: moderate steatosis (33-66%); 3: severe steatosis (>66%)*.* APRI, aspartate transaminase to platelet ratio index; NASH, nonalcoholic steatohepatitis.

**Figure 3 -:**
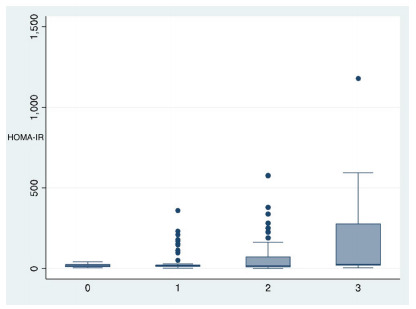
Boxplot analysis for HOMA-IR score and the grade of steatosis determined by biopsy, according to the NASH Clinical Research Network (NASH-CRN) scoring system. 0: normal (<5%); 1: mild steatosis (5-33%); 2: moderate steatosis (33-66%); 3: severe steatosis (>66%). HOMA-IR, homeostatic model assessment for insulin resistance; NASH, nonalcoholic steatohepatitis.

When comparing the multiple AUCs, the null hypothesis of equality of the AUCs^17^ was rejected (p<0.001). Consequently, the APRI AUC and the HOMA-IR AUC were significantly superior to the other scores. Besides, when the test for equality of the APRI AUC and the HOMA-IR AUC was performed, the scores of APRI AUC and HOMA-IR AUC are equivalent (p=0.611). The APRI and HOMA-IR scores according to each steatosis classification are shown as boxplots in Figures 2 and 3, respectively.

The best cutoff point determined by maximum Youden’s J statistics was 0.29 for APRI and 17.11 for HOMA-IR. At these cutoff points, APRI showed high specificity of 82% and low sensitivity of 54%. In contrast, HOMA-IR showed high sensitivity of 84% and low specificity of 48% (see Table 4).

**Table 4 -:** Determination of cutoff points for APRI and HOMA-IR using Youden’s J statistics.

	Cutoff point	Sensitivity (%)	Specificity (%)	Correctly classified (%)	LR+	LR−
APRI	0.29	53.6	81.7	76.2	29.34	0.57
HOMA-IR	17.11	84	48.2	54.8	16.21	0.33

APRI, aspartate transaminase to platelet ratio index; HOMA-IR, homeostatic model assessment for insulin resistance; LR+: positive likelihood ratio; LR−: negative likelihood ratio.

**Figure 4 -:**
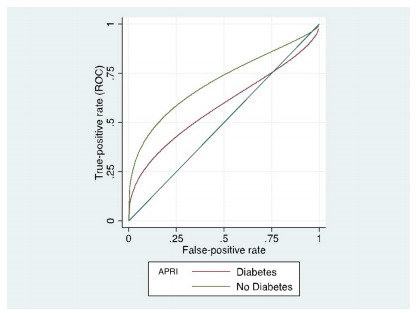
Graph showing the effect of diabetes mellitus on the APRI score diagnostic performance for severe steatosis. APRI, aspartate transaminase to platelet ratio index.

**Figure 5 -:**
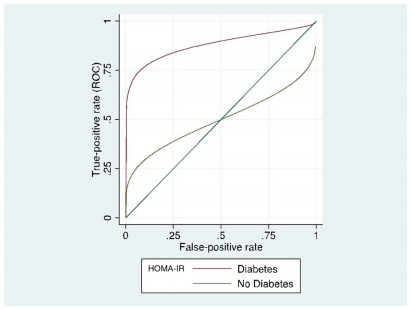
Graph showing the effect of diabetes mellitus on the HOMA-IR score diagnostic performance for severe steatosis. HOMA-IR, homeostatic model assessment for insulin resistance.

Considering high sensitivity of HOMA-IR was related to the high pretest probability, due to the high number of diabetic patients and the known association of steatosis and diabetes or insulin resistance, we performed an ROC regression analysis to assess possible covariates. The results of regression showed that APRI was not affected by the presence of diabetes mellitus (p=0.068), while HOMA-IR was affected (p<0.001) (see Table 5). The effects of diabetes mellitus on APRI and HOMA-IR scores are shown in Figures 4 and 5, respectively.

**Table 5 -:** ROC regression analysis.

ROC model						
	Coef.	Standard error	z	p>|z|	95% CI	
APRI	−0.618	0.339	−1.82	0.068	−1.28	0.05
HOMA-IR	1.276	0.367	3.48	<0.001	0.558	199

APRI, aspartate transaminase to platelet ratio index; CI, confidence interval; HOMA-IR, homeostatic model assessment for insulin resistance; ROC, receiver operating characteristic.

## DISCUSSION

This study involving 143 severely obese patients showed that the use of biochemical-based APRI and HOMA-IR scores could help clinicians predict severe steatosis. HSI, NAFLDS, ALT/AST, and FIB-4 are not useful tools for the evaluation of these patients.

At present, liver biopsy is the gold standard for the diagnosis and stratification of the risk of progression to cirrhosis in NAFLD^1^. However, as it is an invasive procedure and associated with the risk for complications, simpler diagnostic methods need to be investigated. Although infrequent, complications after liver biopsy may occur, and 1-3% of complications will require hospitalization^3^. Pain is the most common complication, but most of the major complications are due to hemorrhage. Bleeding is an especially relevant issue in the steatotic liver, since it is prone to major bleeding^26^. Other reported complications include bacteremia, hemobilia, biliary leak, pneumothorax, and hemothorax^20^.

Imaging methods are the most used noninvasive instruments for the diagnosis of steatosis^26^. Ultrasonography (USG) is the most suitable imaging test due to its cost-effectiveness, but it has limitations, as it requires an important infiltration of adipose tissue before observing the signs of steatosis^26^. Besides, the accuracy reduction with thick panniculus adipose and the dependence on operator and machinery limit their use^26^. Computed tomography (CT) has a sensitivity of 82% and specificity of 100% to diagnose hepatic steatosis when the fat content is more than 30%, but it has the inconvenience of the radiation and limited equipment weight and gantry diameter^26^. Magnetic resonance imaging (MRI) is the most sensitive method to detect increased intrahepatic fat. Unlike USG and CT, it can detect an increase in fat by 3%, but the high cost and low availability limit its use^26^. Transient hepatic elastography (Fibroscan), which is a more sophisticated test, can be used^26^. It is more sensitive to detect lower degrees of steatosis compared with other imaging methods^26^. However, as a disadvantage, the underlying disease, BMI, and type 2 diabetes mellitus can affect the findings and, therefore, should be taken into account in the interpretation of the results^26^. In addition, high cost and limited availability restrict its use^26^.

Biochemical tests for evaluating liver status are especially useful since they are not subject to interexaminer disagreement or subjective analyses. In Brazil, the public healthcare system (SUS) sponsored the bariatric and metabolic surgery (*Ministério da Saúde; Portaria #492;* Brazil), but it lacks a precise guide, not subjected to observer manipulation, for the surgical treatment of severe hepatic steatosis.

Nones et al.^20^ reported that the FIB-4 and NAFLD fibrosis score models had a high negative predictive value (93.48% and 93.61%, respectively) in patients with severe liver fibrosis and concluded that the use of the FIB-4 and NAFLD fibrosis score models in patients with NAFLD allows a diagnosis of severe liver disease to be excluded. Our findings showed these scores in obese patients could not reasonably detect that severe steatosis.

Lee et al.^15^ evaluated the HSI as a screening method for detecting steatosis. However, they used ultrasound as the gold-standard method for liver steatosis, instead of liver biopsy. Also, the study population was composed mainly of lean patients (mean BMI: 24.1 kg/m^2^) different from our sample (BMI: 35-65 kg/m^2^). Kahl et al.^13^ also evaluated nonobese patients, showing high accuracy for detecting steatosis in their tested cohort. All the 92 patients from the cohort of Kahl et al. were nondiabetic^12^.

Diabetes and insulin resistance have a central role in both steatosis installation and its progression to more advanced stages of the disease such as NASH, which makes it the main pathogenic mechanism of NAFLD^24^. Several methods have been used for diagnosing insulin resistance in humans. Glycemic clamp continues to be the gold-standard procedure^16^. However, its complexity limits its application in daily medical practice^16^. Due to its simple determination and calculation method, HOMA-IR has been the most frequently employed technique in both clinical practice and epidemiological studies^24^.

Due to the high pretest probability (36% of the included patients presented diabetes), HOMA-IR showed high sensitivity in detecting severe steatosis in severely obese patients. ROC regression analysis showed that HOMA-IR has limited value for nondiabetic patients. These findings suggest that HOMA-IR is not suitable for helping clinicians to decide the need for an invasive procedure such as bariatric/metabolic surgery. However, insulin resistance and diabetes should be promptly investigated in obese patients. Before these patients manifest any sign of liver disease progression, HOMA-IR could be a good screening method. Considering liver steatosis is a risk factor for HCC^22^, HOMA-IR could be a screening method to identify those patients who should be enrolled in a surveillance program for HCC.

Patients with a high HOMA-IR score should be advised early to adopt a rigorous diet change. Mardinoglu et al.^17^ showed that an isocaloric low-carbohydrate diet with increased protein content in obese subjects with NAFLD leads to downregulation of the fatty acid synthesis pathway and upregulation of folate-mediated one-carbon metabolism and fatty acid oxidation pathways. This diet leads to a dramatic reduction of liver fat resulting from a marked decrease in DNL and an increase in beta-oxidation^17^. Ryan et al.^23^ showed that the Mediterranean diet, a diet high in monounsaturated fatty acids, acts on steatosis and insulin sensitivity, even without weight loss, reduces liver steatosis, and improves insulin sensitivity in an insulin-resistant population with NAFLD, compared with current dietary advice. In an animal model study, exercise training conducted concurrently with the high-fat regimen diet completely prevented the accumulation of lipids into the liver^12^.

The therapeutic approach of NAFLD is currently based on lifestyle intervention, whereas there is no consensus on effective pharmacological treatment. Several drugs (e.g., insulin sensitizers, hepatoprotective agents, and lipid-lowering drugs) have been evaluated as potential therapeutic agents, but the results are inconclusive^11^. Liraglutide is associated with a decrease in BMI^11^. However, a recently published meta-analysis confirmed that liraglutide does not significantly affect hepatic fat content^11^.

Collaborative guidelines from the European Association for the Study of the Liver (EASL), European Association for the Study of Diabetes (EASD), and European Association for the Study of Obesity (EASO) suggest that the data are insufficient for evidence-based recommendations for the use of metformin to treat NAFLD^10^. However, some studies have revealed different results on the benefit of using metformin to treat NAFLD. Metformin could theoretically protect against obesity-associated NAFLD through direct effects on decreasing hepatocyte fat deposition and on inhibiting inflammatory responses in both hepatocytes and macrophages^11^. Liver function tests may ameliorate with metformin, but there is no superiority of metformin compared with dietary treatment on liver histology^11^.

The APRI score was initially used for the evaluation of liver cirrhosis^27^. In a meta-analysis of 40 studies, APRI score greater than 1.0 had a sensitivity of 76% and a specificity of 72% to predict cirrhosis^16^. APRI score greater than 0.7 had a sensitivity of 77% and a specificity of 72% to predict significant liver fibrosis^16^. This study showed that the APRI score has also a high specificity for diagnosing severe steatosis in severely obese patients. Patients with >0.29 APRI score should be considered to a more intense therapy for treating liver steatosis. Thus, the APRI value could support indications for bariatric surgery or metabolic surgery in obese patients.

Medical treatment for weight loss with drugs, diet, exercise, and other lifestyle modification measures may have limited efficacy for some patients, especially in the severely obese ones^28^. Surgical therapy has proven to be very effective for achieving sustained weight loss in the severely obese population^19^ ^,^ ^28^ ^,^ ^30^. Weight loss after bariatric surgery also results in a significant improvement or complete resolution in the metabolic syndrome markers in a vast majority of patients^19^. Sleeve gastrectomy and RYGB are equally effective for treating NAFLD/NASH regarding the histopathological analysis of fat liver content^4^.

This study has some limitations. It is a single-center study and there was a relatively small sample size. Also, obese patients with BMI <35 kg/m^2^ were not included, and certainly some obese patients with BMI <35 kg/m^2^ would benefit from metabolic surgery for the treatment of NAFLD.

## CONCLUSIONS

NAFLDS, FIB-4, AST/ALT, and HSI have no utility for the evaluation of severe steatosis in severely obese patients. Diabetes and insulin-resistance-related biochemical assessment, such as HOMA-IR, can be used as a good screening tool for severe steatosis in such patients. APRI score is the most specific biochemical diagnostic tool for severe steatosis in severely obese patients and can help clinicians to decide the need for bariatric or metabolic surgery.
